# A single nucleotide polymorphism in the UMOD promoter is associated with end stage renal disease

**DOI:** 10.1186/s12881-016-0358-3

**Published:** 2016-12-09

**Authors:** Tingyu Chen, Qianliao Wang, Guisen Li, Li Wang

**Affiliations:** 1 Renal Division and Institute of Nephrology, Sichuan Provincial People’s Hospital, No. 32, West 2nd Duan, 1st Circle Rd., Qingyang District, Chengdu, Sichuan 610072 People’s Republic of China; 2 School of Medicine, University of Electronic Science and Technology of China, No. 32, West 2nd Duan, 1st Circle Rd., Qingyang District, Chengdu, Sichuan 610072 People’s Republic of China

**Keywords:** End stage renal disease, Single-nucleotide polymorphism, UMOD

## Abstract

**Background:**

Several genome-wide association studies revealed that several variants of UMOD gene were related to the estimated glomerular filtration rate (eGFR), CKD or hypertension. In this study, we investigated the association between a common variant rs13333226 in the promoter region of UMOD gene and end stage renal disease (ESRD).

**Methods:**

Variant rs13333226 of UMOD gene was genotyped by using the ABI Real time TaqMan allelic discrimination assay in a case-control study including 638 unrelated patients with ESRD and 366 controls.

**Results:**

The frequency of UMOD SNP rs13333226 GG/GA genotype was significantly higher (36.83% vs. 20.22%, *P* = 4.02 × 10^-8)^ and the frequency of G allele was much higher (19.04% vs. 11.20%, *P* = 4.00 × 10^−6^) in the patients with ESRD than in the controls. The G allele was associated with an increased risk of ESRD (odds ratio 2.30, 95% confidence interval 1.70–3.11, *P* = 6.10 × 10^−8^). And G allele (odds ratio 2.33, 95% confidence interval 1.32–4.13, *P* = 3.65 × 10^−3^) was associated independently with ESRD.

**Conclusions:**

A common variation rs13333226 in the promoter region of UMOD gene was independently associated with ESRD in Han Chinese.

## Background

End stage renal disease (ESRD), was one of the most serious principal challenges and resulted in a serious public health and financial burden worldwide. In addition, ESRD caused high morbidity and was an independent risk factor for cardiovascular disease or overall mortality in patients with chronic kidney disease (CKD) [1, 2]. It affected over 0.03% of the Chinese adult population [3].

Multiple factors were involved in the progression of kidney diseases into ESRD, and the development of ESRD was the result of interaction between genes and environment [4–7]. Familial clustering phenomenon was common in patients with ESRD [4, 5, 7, 8]. A recent study reported that a CKD patients, who had a first-degree relative with ESRD, significantly increased the relative risk of developing ESRD [8]. Polymorphisms of many genes were reported to be associated with ESRD, such as ACE, APOL1, and MUC1 [7].

Recently, several genome-wide association studies (GWAS) showed that lots of genetic risk loci (such as: rs12917707, rs4293393, and rs6497476) were associated with estimated glomerular filtration rate (eGFR), CKD or hypertension [9–13]. Most studies consistently showed that the common variant rs12917707 of the UMOD gene have been strongly associated with eGFR, prevalent and incident CKD and ESRD in general population cohorts [9, 11, 14]. The relationship between T allele of rs12917707 and lower risk of CKD and ESRD was confirmed in other two case-control studies [15, 16]. These results suggested that the variants of UMOD gene contributed to the genetic susceptibility to ESRD. But a study recently reported that the association between rs12917707 and GFR was not observed in Italian diabetic patients [17]. And the minor allele frequency of rs12917707 (T allele) was only 1% in Han Chinese (http://hapmap.ncbi.nlm.nih.gov/index.html.en). Another single nucleotide polymorphism (SNP) in the promoter region of UMOD gene, rs13333226, was reported to be associated with hypertension, and serum uric level [18, 19]. The G allele carriers had higher diastolic blood pressure and higher plasma uric acid compared with A/A homozygotes [18, 19]. In this study, we explored the association between rs13333226 and the susceptibility to ESRD.

## Methods

### Patients and clinical data

Total 638 unrelated Han Chinese patients with ESRD were recruited from southwest of China. The diagnostic criteria of ESRD were eGFR less than 15 or have received dialysis (hemodialysis or peritoneal dialysis). The patient who had acute kidney injury was excluded. The average age of their onset of ESRD (CKD 5 or 5D) was 54.1 ± 16.0 years old (from 14 to 87 years old). The primary causes of ESRD included chronic glomerulonephritis (CGN), diabetic kidney disease (DKD), hypertensive nephropathy (HTN), as well as other causes. Three hundred and sixty-six healthy individuals served as controls. All the controls didn’t have hypertension, diabetes, as well as acute or chronic kidney diseases. The average age of the controls was 52.9 ± 13.3 years-old (from 18 to 87 years old). The protocol was approved by the Ethics Review Committee of Sichuan Provincial People’s Hospital. Informed consent was signed by all subjects.

### DNA extraction and genotyping

DNA was extracted from peripheral blood collected from patients with ESRD and healthy controls by using ‘salting-out’ method. The genotyping was conducted by real–time PCR. The primers and probe were obtained from Applied Biosystems (ABI, Foster city, CA). Genotyping of the rs13333226 SNP in the UMOD gene was performed using an ABI 7900 Real-time System (ABI, Foster city, CA). Assays were run at a final volume of 25 μl consisting of 12.5 μl of TaqMan®Gene Expression Master mix (ABI, Foster city, CA), 7.5 μl of primers (400 nM both forward and reverse) and probe (200 nM) (final concentration), 5 μl of input target DNA. Genotype clustering and calling were performed using SDS 2.3 Software (ABI, Foster city, CA).

### Statistical analyses

Deviation from Hardy-Weinberg equilibrium was tested in healthy controls. Comparison of genotype and allele frequency between patient group and control group was done by *X* ^2^ test. Genotype-phenotype associations were tested under dominant as well as recessive genetic model. The effect of genotype on ESRD was also investigated with logistic regression analysis. Statistical analyses were performed using SPSS 17.0 software package (SPSS Inc., Chicago, IL). Statistical power was calculated by a software “PS: Power and Sample Size Calculation” [20]. The SNP data of UMOD gene derived from 1000 Genomes project (GRCh38) CHB (Chinese Han Beijing) + CHS (Chinese Han South) were used to perform pairwise linkage disequilibrium analysis between rs13333226 and the previous GWAS hit rs12917707, rs4293393, as well as rs6497476 by Haploview software [21].

## Results

The percentage of male in the cases was much higher than in the controls (55.8% vs. 33.9%, *P* = 2.20 × 10^−11^). The demographic and clinical data were listed in Table 1. There was no deviation from Hardy-Weinberg equilibrium in controls (*P* = 0.073). The coefficient (r^2^) for pairwise linkage disequilibrium (LD) between rs13333226 and rs12917707, rs4293393, as well as rs6497476 were 0.099, 1, 0.883, respectively.

**Table 1:** Demographic and clinical characteristics of the participants

	Cases (*n* = 638)	Controls (*n* = 366)	*P* values
Male, *n* (%)	356 (55.8%)	124 (33.9%)	2.20 × 10^−11^
Age (y)	54.1 ± 16.0	52.9 ± 13.3	0.13
Primary causes of ESRD			
Chronic glomerulonephritis	250 (39.2%)	-	-
Diabetic kidney disease	100 (15.7%)	-	-
Hypertensive nephropathy	87 (13.6%)	-	-
Other causes	95 (14.9%)	-	-
Unknown	106 (16.6%)	-	-

Compared to the healthy controls, the frequency of UMOD SNP rs13333226 GG/GA genotype was significantly higher (36.83% vs. 20.22%, *P* = 4.02 × 10^-8)^ and the frequency of G allele was much higher in the patients with ESRD (19.04% vs. 11.20%, *P* = 4.00 × 10^−6^). The frequencies of GG/GA genotype and G allele were also much higher in ESRD patients with diabetes mellitus, without diabetes mellitus, or with hypertension (Table 2). For male individuals, the frequency of GG/GA genotype was much higher in patients than in controls (35.4% vs. 21.8%, *P* = 5.10 × 10^−3^), and for female individuals, the difference was significant (38.4% vs. 19.7%, *P* = 2.00 × 10^−6^).

**Table 2:** The frequencies of genotypes and alleles of rs1333326 in cases and controls groups

	Genotype frequency			Allelic frequency	
	GG	AG	AA	G	A
Control	8	66	292	82	650
All cases	8	227	403	243	1033
	X^2^ = 35.08, *P* =2.41 × 10^−8^			X^2^ = 21.09, *P* =4.00 × 10^−6^	
Cases with DM ^a^	2	40	58	44	156
	X^2^ = 19.79, *P* =5.00 × 10^−5^			X^2^ = 15.67, *P* =7.60 × 10^−5^	
Cases without DM ^a^	6	187	345	199	877
	X^2^ = 30.96, *P* =1.89 × 10^−7^			X^2^ = 17.65, *P* =2.70 × 10^−5^	
Cases with hypertension ^a^	7	202	360	216	922
	X^2^ = 33.69, *P* =4.84 × 10^−8^			X^2^ = 20.12, *P* =7.00 × 10^−6^	

^a^Compared to controls

The G allele was associated with higher risk of ESRD in dominant model [odds ratio (OR) = 2.30, 95% confidence interval (CI): 1.70–3.11, *P* = 6.10 × 10^−8^] with a statistical power of 1.00, in an additive model (OR = 1.96, 95%CI: 1.48–2.60,*P* = 2.00 × 10^−6^), but not in recessive model (OR = 0.57, 95% CI: 0.21–1.53, *P* = 0.26). The G allele was significantly associated with ESRD in dominant model in the subgroup of patients without diabetes mellitus (*N* = 538, OR = 2.21, 95% CI: 1.62–3.01, *P* = 5.55 × 10^−7^), with diabetes mellitus (*N* = 100, OR = 2.86, 95% CI: 1.78–4.58, *P* = 1.30 × 10^−5^), as well as with hypertension (*N* = 569, OR = 2.29, 95% CI: 1.69–3.11, *P* = 1.19 × 10^−7^).

The effect of genotype on ESRD was also investigated by a multivariable logistic regression analysis including age of initial ESRD, sex, hypertension and diabetic mellitus. The result showed that male (OR = 2.05, 95% CI: 1.19–3.51, *P* = 9.31 × 10^−3^) and G allele at rs13333226 (OR = 2.33, 95% CI: 1.32–4.13, *P* = 3.65 × 10^−3^) was independently associated with the susceptibility to ESRD.

## Discussion

UMOD gene encodes Tamm-Horsfall protein, also known as uromodulin, which is a glycoprotein exclusively synthesized by the thick ascending limb (TAL) and early distal convoluted tubule in the kidney [22]. Lots of studies revealed that the variants of UMOD were associated with different risk of CKD, cardiovascular disease, hypertension, and hyperuricemia, T allele of rs12917707 was associated with lower risk of CKD and ESRD [9, 10, 18, 19, 23, 24]. But the relationship between variants of UMOD and risk of kidney disease was complicated, the several rare mutation in UMOD gene has been described as a cause of uromodulin-associated kidney disease, an autosomal dominant disease [25, 26]. Prudente et al. recently demonstrated that the previously reported strong association between rs12917707 and GFR in diabetic patients from Sweden was not observed in Italian diabetic patients [17]. It suggested that there was a heterogeneous effect across the two different samples [17]. In this study, we investigated the association between a promoter SNP rs13333226 of UMOD and susceptibility to ESRD.

Our results indicated that the frequency of rs13333226 GG/GA genotype and G allele was significantly higher in the patients with ESRD, compared to healthy controls. The G allele was associated with higher risk of ESRD in dominant model. G allele and male was associated independently with the susceptibility to ESRD by multivariate logistic analysis. This relationship still existed after adjusted by sex, age, as well as presence or absence of diabetes mellitus and hypertension. Our results demonstrated that rs13333226 didn’t correlate tightly with rs12917707 in Chinese Han population. It suggested that the effect of rs13333226 on CKD or ESRD was different from rs12917707. A previous case-control association study showed that G allele of rs13333226 was independently associated with a decreased risk of nephropathy, and the G allele was also associated with a higher eGFR and lower systolic blood pressure in the study cohort [27]. However, two other studies have shown that G allele carriers have higher diastolic blood pressure and higher plasma uric acid compared with A/A homozygotes [18, 19].

The G allele at rs13333226 and C allele at rs6497476 were demonstrated to increase transcriptional activity in HEK-293 cells and mlMCD3 cells by luciferase reporter assay [19]. The rs13333226 G allele carriers have higher plasma uric acid than A/A homozygotes [19]. Higher serum uric acid was reported to associate significantly with increased risk for kidney functional impairment and SUA ≥6.0 mg/dL was a significant risk factor for rapid decline in eGFR [28]. In a prospective children and adolescents cohort study, those with higher uric acid levels (≥5.5 mg/dL) had shorter times to renal outcomes by a multivariable parametric time-to-event analysis [29]. Trudu et al. reported that two UMOD risk variants (G allele of rs12917707 and T allele of rs4293393) increased UMOD expression which led to salt-sensitive hypertension and to the presence of age-dependent renal lesions in transgenic mice [24]. High expression of uromodulin could activate the renal sodium cotransporter NKCC2 which induced hypertension in UMOD transgenic mouse model [24]. UMOD has also proved to regulate the renal outer medullary potassium channel, thus increasing salt reabsorption along the TAL [30]. It could be speculated that the G allele at rs13333226 could increase carriers’ risk of ESRD by enhancing UMOD gene expression, increasing TAL salt reabsorption and serum uric acid levels, as well as increasing risk of hypertension. We haven’t detected serum sodium or uric acid levels in these patients, because the two parameters were influenced by many factors in patients with hemodialysis.

Although there was an independent association between G allele at rs13333226 and ESRD, we still need direct functional evidence to illustrate that whether the G allele at rs13333226 was a causal variant. And a prospective cohort study with large sample size would provide more solid evidence.

## Conclusions

In summary, our data indicates that common genetic variation rs13333226 in the promoter region of UMOD gene was independently associated with ESRD in Han Chinese.

## Abbreviations

CKD: Chronic kidney disease
eGFR: Estimated glomerular filtration rate
ESRD: End stage renal disease
SNP: Single nucleotide polymorphism
UMOD: Uromodulin
